# The Behavior of Cement-Bonded Particleboard with Modified Composition under Static Load Stress

**DOI:** 10.3390/ma14226788

**Published:** 2021-11-10

**Authors:** Tomas Melichar, Jiri Bydzovsky, Richard Dvorak, Libor Topolar, Sarka Keprdova

**Affiliations:** 1 Institute of Technology of Building Materials and Components, Faculty of Civil Engineering, Brno University of Technology, 602 00 Brno, Czech Republic; bydzovsky.j@fce.vutbr.cz (J.B.); keprdova.s@fce.vutbr.cz (S.K.); 2 Institute of Physics, Faculty of Civil Engineering, Brno University of Technology, 602 00 Brno, Czech Republic; dvorak.r1@fce.vutbr.cz (R.D.); topolar.l@fce.vutbr.cz (L.T.)

**Keywords:** cement-bonded particleboard, modification, composition, adverse environment, frost, stress, static load, acoustic emission, defect analysis

## Abstract

This article presents research on the behavior of cement-bonded particleboards under mechanical stress caused by the static load. The composition of the boards was modified using alternative raw materials–dust (DU) forming during the processing of cement-fibre boards and particle mixture (PM) generated in the production of cement-bonded particleboards. The particleboards (1-year-old) were subjected to an adverse environment (100 to 250 frost cycles). Mechanical parameters were tested, and the development of defects during static load of the boards by bending was analyzed using acoustic emission. Particleboards with modified compositions are slightly more resistant to adverse environments. The results of the acoustic emission showed the different types of defects occurring under stress by bending. Standard-composition particleboards showed defects located mainly under the cylindrical stress-test head. The modified boards showed larger location distribution of the occurring defects that were also concentrated further away from the cylindrical stress head. The energy during the occurrence of defects was higher in the modified boards in the location of weight application than in the reference boards.

## 1. Introduction

Cement-bonded particleboards are a frequently used construction element. This material combines the characteristics of the cement matrix and spruce chips. Over 55,000 m^3^ of these boards are produced annually in the Czech Republic. The boards are used in many construction units—façades, floors, partitions and railings, etc. Annually, approximately 12,000 t of by-products are created during the processing of these boards without further use, ending in landfills. Specifically, approx. 7000 t/year of dust is produced (from sanding and cutting the boards), and approx. 5000 t/year of edgings (from formatting the boards).

The current environmental situation strengthens efforts leading to the development of waste-free technologies. In the case of existing production technologies, the producers make efforts toward maximizing the use of whatever waste they produce. Therefore, two modified mixtures of cement-bonded particleboards were designed and developed in collaboration with the Czech producer CIDEM Hranice, a.s. Among other sources, the composition of these formulae reflects the existing findings of the authors [1]. Findings exist from a number of studies focused on the use of various alternative materials in cement-bonded particleboards, for example [2,3,4,5,6,7,8,9,10,11,12,13,14,15,16,17,18,19,20,21,22].

However, no research was conducted regarding the re-use of by-products created by the production of cement-bonded particleboards in their further production. Only Ezerskiy et al. [23] focused on the use of waste from cement-bonded particleboard production in fine-grain concrete. However, the Ezerskiy study mainly regards the proportion of formulae depending on the bulk weight and strength characteristics of the designed concrete. Thus, there was no emphasis on the study of links from the material perspective of the proposed mixtures. Instead, the focus remained on calculating mathematical models evaluating the achieved characteristics of the designed materials with modified composition. Another important fact is that Ezerskiy et al. used waste produced by a company named TAMAK. Considerable differences are apparent from the comparison of the weight composition of the cement-bonded particleboards of those produced by CIDEM (cement—50%, wood—18%, water—30%, hydration additives—2%) and TAMAK (cement—65%, wood—24%, water—8.5%, hydration additives—2.5%).

Thus, in the study presented here, primary components of the matrix and filler of the cement-bonded particleboards were substituted with by-products [24,25,26,27], specifically those from the cement-bonded particleboard production (produced by CIDEM Hranice, a.s.). The board composition was modified by dust (DU) and particle mixture (PM) from the cement-bonded particleboard production.

A more notable degradation occurs in materials on the basis of a cement matrix and an organic filler (mainly wood) due to temperature and humidity changes (see, for example, findings of studies [12,13]) than in the case of inorganic construction materials (concrete, mortar, etc.). The key criterion for the usability of board materials in constructions is their bending strength and modulus of elasticity in bending. Thus, it may be assumed that boards are mostly stressed by the bending load. The behavior is, therefore, essential of cement-bonded particleboards (exposed to adverse environment) over the course of static flexural (bending) load. The key is a clear identification, location and description of occurring defects, ultimately leading to the definitive weakness of the material.

It was found that the subject of long-term durability of boards with modified composition was not sufficiently covered by other authors. Research of expert publications also showed the absence of findings regarding the localization of the defects in cement-bonded particleboards with a modified composition. Some authors focus on using the acoustic emission method for the analysis of a wide variety of construction materials, for example [28,29,30,31,32,33,34,35,36,37,38,39,40,41,42,43]. However, the currently available publications offered no mention of evaluating the behavior of cement-bonded particleboards using the acoustic emission method during their mechanical stress by static bending.

Thus, the localization of the defects in the cement-bonded particleboards within this study was analyzed in detail through a combination of physical, mechanical, microstructural and acoustic techniques. Boards aged one year were subjected to testing and analysis upon reaching 100 to 250 frost/defrost cycles. During the stress test of the bending load, it was possible to describe processes of formation and progress of active defects in the structure of the cement-bonded particleboards thanks to the synchronized application of acoustic emission and an electro-hydraulic device (with the possibility to monitor and record data on a PC). Precious is the description of differences in the behavior of boards of different compositions, as well as the comparison of the effects of the adverse environment. Furthermore, also essential was the supplementation of other techniques, such as optical microscopy.

## 2. Materials

The cement-bonded particleboards for these tests were produced in collaboration with CIDEM Hranice, a.s. The default formula of their boards consists of cement, spruce chips, sodium silicate and aluminium sulfate. The composition of the cement-bonded particleboards is shown in the following table (see Table 1).

The composition of modified formulae is based on findings and existing results of research by the authors in collaboration with CIDEM Hranice, a.s., see for example [1,26,27]. These results, thus the material for the composition of the modified formulae, were obtained within long-term cooperation of the authors with the Institute of Technology of Building Materials and Components, Faculty of Civil Engineering, BUT and CIDEM Hranice, a.s. The amounts of modifying components also reflect the current production of by-products at the CIDEM Hranice, a.s. production line.

**Figure 1:**
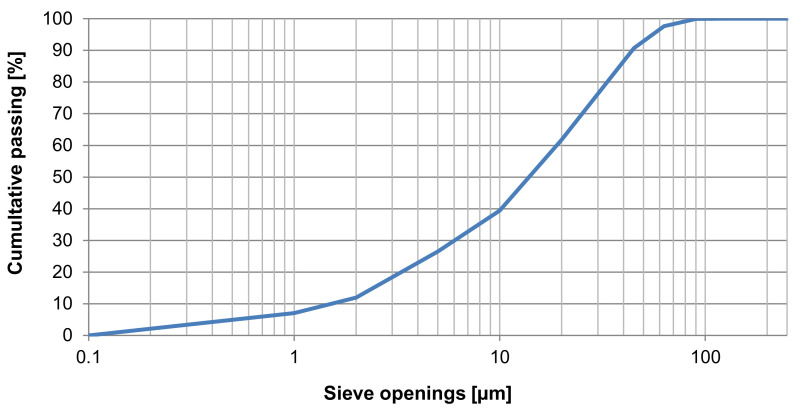
Analysis results of size and distribution of particles in the CEM II/A-S 42,5 R cement.

Portland-slag cement CEM II/A-S 42,5 R whose specific surface area of 458 m^2^/kg (for size and distribution of particles–see Figure 1) and density of 3124 kg/m^3^ was used for the production. The initial setting time ranges between 215–250 min. Standardized compressive strength after 28 days reaches values of approx. 59 MPa. The chemical composition of the cement is shown along with the by-products DU and PM in the following table (see Table 2). Measurement methods used to determine the chemical composition were as follows: gravimetric, photometric, flame atomic absorption spectrophotometry, complexometric titration and mercurymetry (in an external laboratory).

**Figure 2:**
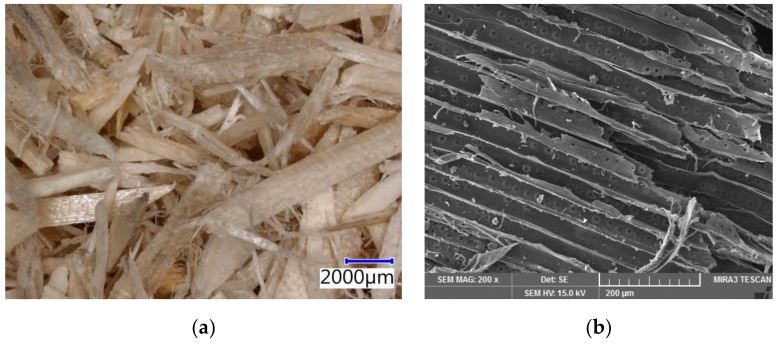
Spruce chips used in cement-bonded particleboard production—pictures from Keyence VHX-950F optical microscope and Tescan MIRA3 XMU scanning electron microscope: (**a**) structure of chips—optical microscope; (**b**) structure of spruce chips in detail—SEM.

**Figure 3:**
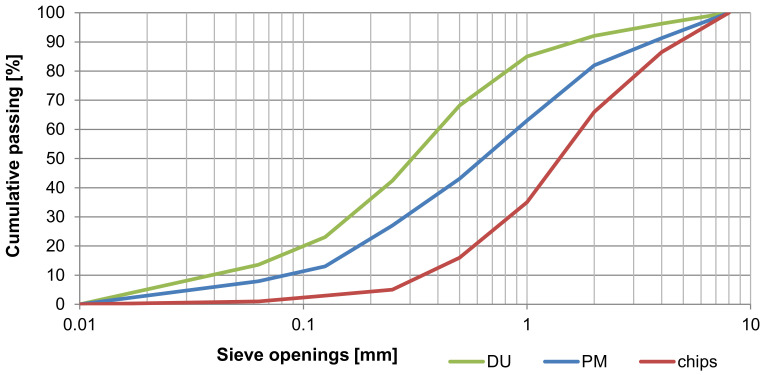
The results of size and particle distribution analysis of alternative raw materials DU and PM, including the comparison with the primary spruce chips.

The particleboards are based on spruce wood (see Figure 2). The figure below shows the distribution and size of chips along with alternative compounds (sieve analysis; see Figure 3). Additionally, water and hydration compounds were used in the production of these boards.

**Figure 4:**
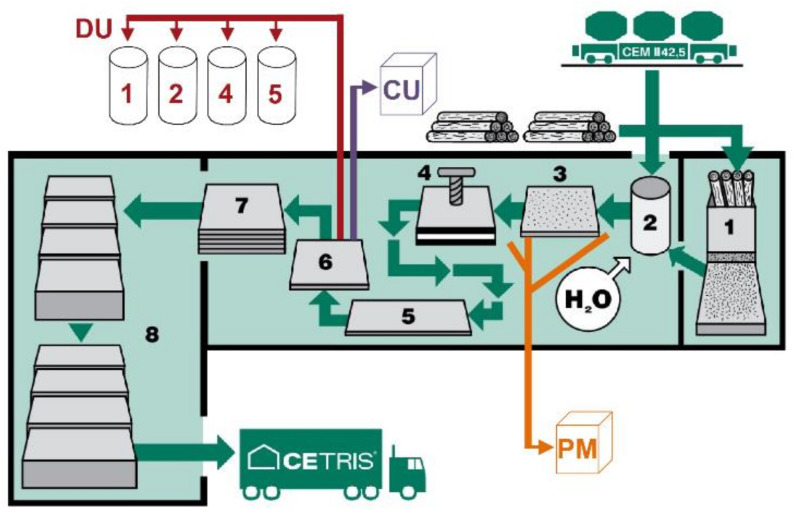
Production flow-chart of CIDEM Hranice, a.s cement-bonded particleboard: 1—spilling; 2—preparation of mixture; 3—layering of boards; 4—pressing; 5—drying; 6—formatting; 7—storage; 8—transport (red DU 1, 2, 4, 5—silos for collecting the dust; violet CU—covered space for collecting cuttings from formatting particleboards; orange PM—covered space for storing the particle mixture) [44].

From the perspective of collecting by-products, the cement-bonded particleboard production line has a total of four silos (Towers 1, 2, 4 and 5; see Figure 4). These towers capture and temporarily store particle by-products. The modification of the formula composition was created using the dust from grinding DU formed as a by-product of processing these boards. DU is collected as the by-product in Tower 2. The dust-removal Tower 2 is designed to collect dust from the formatting saw. The dust from the formatting saw is extracted via cyclone device, where larger particles are collected, while the dust continues to the textile filter of the dust-removal tower. The variability of the properties (thus also the composition) of DU is lowest among all the by-products (see other dust-removing towers), practically nearly constant. Therefore, DU could be considered a potentially very interesting product for further use.

Additionally, particle mixture PM remaining after the production of cement-bonded particleboards was used. This by-product is formed, for example, during the change of production conditions (adjustment of board thickness, mixture, etc.; see Figure 4). PM is not stored in towers but in a covered space along with cuttings (from cutting cement-bonded particleboards).

Detailed analysis results for the by-products from processing and production of cement-bonded particleboards (produced by CIDEM Hranice, a.s.) are covered by Melichar et al. in [24,25,26,27]. The DU and PM alternative components have notably different grain (see Figure 3), where these materials show a grain range from 125 µm to 2 mm. The DU material features a higher ratio of smaller particles. This fact would be expected given the origin of the individual components (DU—particles from cutting, PM—a fresh mixture for the production of cement-bonded particleboards). The following images show the DU and PM by-products used as alternative components of cement-bonded particleboards.

The chemical composition in terms of the presence of the individual compounds corresponds to cement-bonded particleboards (see Table 2). The fact that DU contains a higher amount of wood material is significant. The wood content is established using the TOC method (total organic carbon content—a calculation with comparative samples of cement and primary chips). The difference in the amount of other chemical compounds in comparison with the DU and PM are not significantly notable, with the exception of the SiO_2_ content. With respect to the mineralogical composition, it may be stated that in both these cases of alternative materials, the substances are on the basis of a silicate matrix. Therefore, the following mineralogical phases were identified: portlandite, quartz, calcite, traces of cement clinker minerals (particularly belite), as well as ettringite and a slightly increased background (characterizing the presence of the amorphous phase—in this case hydrating admixtures, possibly the amorphous part of cellulose).

Additionally, the absorption rate was established in the case of alternative DU and PM components. It was found that PM shows an absorption rate of 38.6% and DU an absorption rate of 50.8%. This corresponds with the identified content of wood matter and granularity. The DU and PM materials were used for the production of the cement-bonded particleboards in a raw state, meaning without further mechanical or other modification. On the contrary, Ezerskiy et al. [23] focused on the possible use of CBPB waste in a modified composition, specifically, grinding in a ball mill for 30 min.

**Figure 5:**
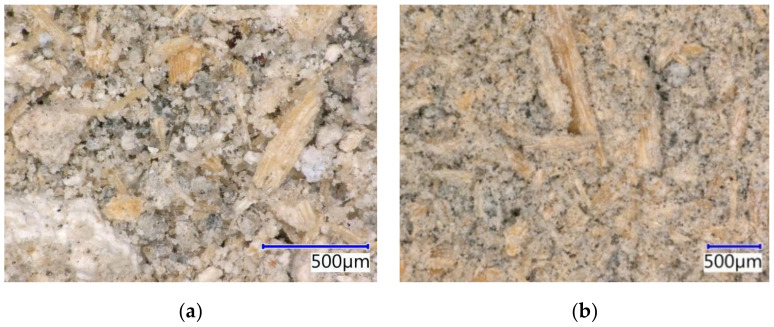
Alternative components (by-products) for modification of cement-bonded particleboard composition—images from Keyence VHX-950F optical microscope: (**a**) PM—particle mixture from cement-bonded particleboard production; (**b**) DU—dust from cutting of cement-bonded particleboards during their formatting.

**Figure 6:**
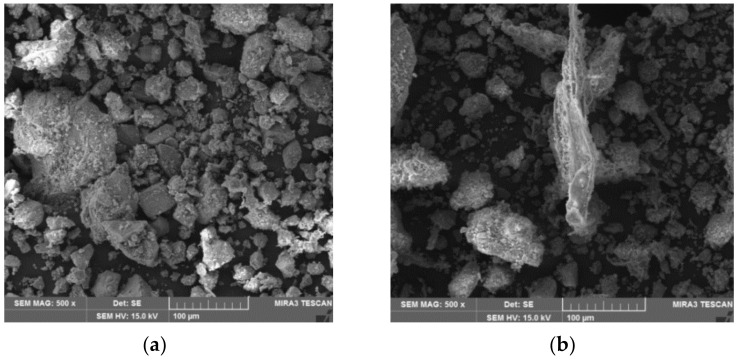
Alternative components (by-products) for modification of cement-bonded particleboard composition—pictures from TecScan MIRA3 XMU scanning electron microscope: (**a**) PM—particle mixture from cement-bonded particleboard production; (**b**) DU—dust from cutting of cement-bonded particleboards during their formatting.

Furthermore, the structure, specifically the microstructure of PM and DU, was analyzed in detail. First, analyses using the optical microscope were executed (see Figure 5), followed by a scanning electron microscope (see Figure 6). Both the alternative components are cases of particulate matter where the size of particles corresponds to the results establishing granularity (see Figure 3). Thus, DU contains a larger number of smaller particles, as well as more wood matter. PM has larger clusters of particles (larger grain) formed during the ageing of the mixture during outdoor storage. Both PM and DU contain a very mature cement matrix. The considerable amount of chips is covered by a fine layer of the matrix, on which the hydration products of cement are also visible.

All primary materials were batched into the mix (directly in the CIDEM Hranice, a.s. production facility) automatically. This is the standard method of dosing and mixing compounds in the industrial production of cement-bonded particleboards. Only the alternative compounds were manually added directly to the mixer in a defined amount. Boards covering 4 mixing devices were produced for each formula (a total of approximately 11 m^3^ of mixture for board production). Test specimens were then prepared from the second and third mixer load to ensure sufficient homogeneity of the required formula, specifically the mixture. Due to the length of the production line, a transitional modification of the composition of the cement-bonded particleboards may occur prior to achieving the required consistent composition of the mixture.

## 3. Methods

The production of test specimens took place directly at the CIDEM Hranice, a.s. production line. This company is a leading producer of cement-bonded particleboards in the Czech Republic with a significant level of export abroad. The test specimens were cut from the boards approximately ten days after production. The specimens were transported to the laboratories of the Institute of Technology of Building Materials and Components, Faculty of Civil Engineering, BUT, where they were prepared for all planned tests and analyses.

### 3.1. Curing of Test Specimens

The test specimens were stored in the laboratories of the Institute of Technology of Building Materials in an environment with a relative humidity of 75% at a temperature of approximately 22 °C. The relevant technical regulations set no conditions for storing cement-bonded particleboards for long-term maturing of the test specimens. The reason for establishing the above conditions was the fact that these boards contain a relatively high percentage of wood matter (approximately 63%). Despite being mineralized, this wood matter changes its percentage in response to changes in humidity which could have (in the case of excessive humidity—for example, submersion in water) a negative effect on the course of maturing.

On the other hand, the possible storage of boards in an environment with low humidity would not provide sufficient hydration of the cement matrix. The relative humidity in the place in the storage of these boards was monitored once every two months. The fluctuation in humidity and temperature over the course of their maturing did not exceed ±4% and ±3 °C. The specimens were left in these conditions for a period of one year in order to evaluate their parameters in a more long-term view. The specimens exposed to adverse conditions prior to testing were removed from the storage conditions earlier. The timing was planned to ensure that all specimens (both reference specimens and those exposed to frost) were tested at the same age, meaning one year.

### 3.2. Adverse Environment—Frost/Defrost Cycles

**Figure 7:**
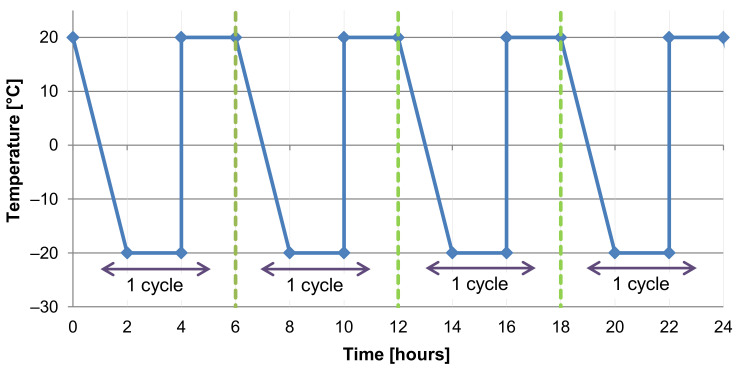
Temperature course of frost/defrost cycles; 4 cycles—24 h; phase 0–4 h of one cycle takes place without the presence of water, and during the final phase of the cycle 4–6 h, the testing chamber is filled with water, where the test specimens are submerged at (20 ± 2) °C.

For both the Czech Republic and most of Europe, a typical example of an adverse environment is the cyclical frost/defrost effect. Thus, the focus was aimed at monitoring the effect specifically from this environment. The testing of frost resistance of cement-bonded particleboards is covered by the technical standard EN 1328 [45]. Before testing, test specimens are submerged in a water bath at the temperature of 20 °C for 48 h. Subsequently, fluctuating frost/defrost takes place with the presence of water. The course of temperature cycles in relation to time is shown in the following graph (see Figure 7).

The EN 1328 standard requires a minimum of 50 of the above-described frost/defrost cycles. The evaluation of frost resistance that characterizes durability was executed within the range of 100 to 250 cycles. The reason was the reliable evidence of changes in properties of the modified boards in a more long-term horizon. Therefore, the following sets of test specimens were produced and exposed to the adverse environment:

- A set of reference specimens/Ref—stored under the conditions covered in Section 3.1;
- A set of specimens exposed to 100 frost/defrost cycles/M10;
- A set of specimens exposed to 150 frost/defrost cycles/M15;
- A set of specimens exposed to 200 frost/defrost cycles/M20;
- A set of specimens exposed to 250 frost/defrost cycles/M25.

Each set of cement-bonded particleboards for establishing one parameter consisted of 6 test specimens.

Therefore, it is apparent that from the perspective of unifying the age of testing, the specimens exposed to 250 frost/defrost cycles had to be removed from the storage of 75% humidity approximately after 10 months of maturing. The required number of cycles of exposure to the adverse environment was executed over the course of approximately two months. At the age of 1 year, the specimens were ready for evaluation of their properties, including the analysis of the localization of defects resulting from flexural bending load.

### 3.3. Physical and Mechanical Tests

The requirements for the properties of cement-bonded particleboards are stipulated in standards [46,47,48,49]. This standard also establishes the requirements for additional properties, such as size changes, resistance against puncture and frost resistance. Before testing, the test specimens were always stored in the defined conditions, meaning at the relative humidity of (65 ± 5)% and temperature of (20 ± 2) °C. All measurements and tests were performed upon the specimens reaching a constant weight in this environment. In this case, the constant weight is defined by the difference in two consecutive weight tests in the interval of 24 h, where this difference is ≤ 0.1%.

#### 3.3.1. Density

The density of the boards was established in accordance with the requirements of the technical standard EN 323 [50]. Density is determined as the ratio of the weight of the test specimen to its volume. Determining both the volume and weight were executed at the same humidity of the test specimens. The test specimen for this test has the shape of a square with a minimal side length of 50 mm. Additionally, specimens used for testing bending characteristics were used for establishing the density of analyzes boards. Density is stated in kg/m^3^.

#### 3.3.2. Bending Strength and Modulus of Elasticity in Bending

Strength and modulus of elasticity in bending were established in accordance with the relevant technical EN 310 standard [51]. Bending characteristics are established by applying a load into the center of the test specimen. The specimen is placed on two cylindrical supports, and the load is applied using a cylindrical weight head located parallel with the supports between them, thus creating a three-point bend. The elasticity module is established using the linear part of the load curve. The bending strength of each specimen is calculated by stipulating the ratio of the bending moment M at maximum load F_max_ to the moment of its entire cross-section.

The width of the test specimens is 50 mm, and the length is established as 20 times thickness + 50 mm. The minimum length of the specimen is 150 mm, and the maximum is 1050 mm.

**Figure 8:**
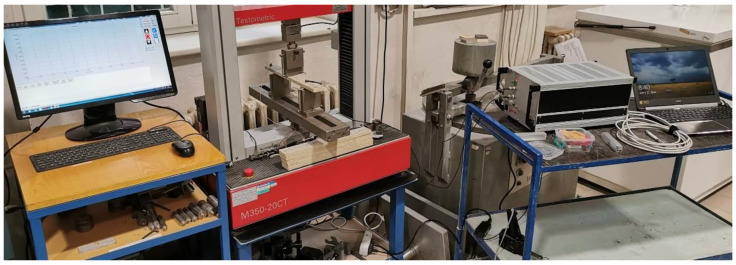
The scheme of arrangement for establishing bending strength and elasticity modulus with continual monitoring and recording of defects using acoustic emission; left-to-right: the press including computer technology, the testing specimen in the press with an attached exciter and acoustic emission sensor, the device for the processing of acoustic emission signal, including computer technology.

**Figure 9:**
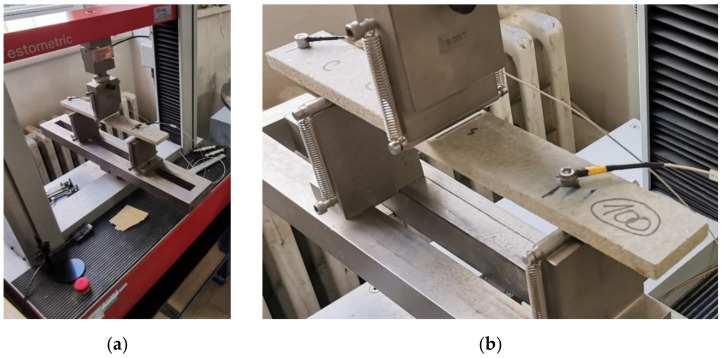
The scheme of arrangement for establishing the bending strength and elasticity modulus with continual monitoring and recording of acoustic emissions: (**a**) testing arrangement; (**b**) a detail of the tested specimen with placed exciter and acoustic signal sensor.

The distance between the centers of the supports is established with the precision of 1 mm 20 times the nominal thickness of the board. The load is established at a constant rate to achieve the maximum, meaning the damage of the specimen, within (60 ± 30) s. The testing scheme arrangement is shown in Figure 8 and Figure 9.

In this specific case, the thickness of the boards was 12 mm, and the distance between the supports was 240 mm. With respect to the analysis of the localization of defects, the length of the test specimens was determined to be 380 mm. Thus, an additional 45 mm were left on each side of the specimens for the placement of the acoustic emission sensors. The rate of establishing the bending load was 3 mm/min, thus resulting in the damage of all tested specimens within 35 to 50 s.

Bending strength f_m_ (N/mm^2^) is determined according the following equation [51]:

f_m_ = (3 × F_max_ × l_1_)/(2 × b × t^2^). (1)

Modulus of elasticity in bending E_m_ (N/mm^2^) is determined according the following equation [51]:

E_m_ = [l_1_^3^ × (F_2_ − F_1_)]/[4 × b × t^3^ × (a_2_ − a_1_)], (2)

where: l_1_ is the distance between the centers of the supports in mm; b is the width of the test specimen in mm; t is the thickness of the test specimen in mm; F_2_ − F_1_ the increase in the load in the linear part of the load curve in N; F_1_ is approximately 10% and F_2_ approximately 40% of the maximum load F_max_; a_2_ − a_1_ the increase in bend in the center of the length of the testing specimen (corresponding to F_2_ − F_1_).

#### 3.3.3. Transverse Tensile Strength Perpendicular to the Plane of the Board

The tensile strength perpendicular to the plane of the board was established according to the technical EN 319 standard [52]. According to the provisions of this norm, the characteristic is established upon tensile load affecting the tested specimen to the point of its damage in the direction perpendicular to the plane of the specimen (identical to the plane of the field). Tensile strength perpendicular to the plane is determined from the maximum strength affecting the surface for the tested specimen.

The test specimens for this testing have the shape of a square with the side of (50 ± 1) mm. The specimens must be cut precisely, with a 90° angle, and their edges must be straight and clean.

To enable their attachment into the testing electro-hydraulic device, blocks are glued onto the specimens. Steel targets fixed by an adhesive on the basis of a two-component epoxy resin (the glue solidifies approximately 24 h) were used for this purpose. As in the case of bending, the rate of the load of the specimens must be established to achieve damage within (60 ± 30) s. The rate for establishing the tensile strength was 3 mm/min, thus reaching damage in all tested specimens between 30 to 40 s.

Transverse tensile strength perpendicular to the plane of the board f_t┴_ (N/mm^2^) is determined according to the following equation [52]:

f_t┴_ = F_max_/a × b, (3)

where: F_max_ is the load affecting the testing specimen at the moment of damage in N; a the length of the testing specimen in mm; b the width of the testing specimen in mm.

### 3.4. Localization of Defects—Acoustic Emission

The acoustic emission (AE) activity was monitored throughout the three-point bending test. Acoustic emission is among the most modern methods for evaluating materials in engineering and fatigue applications. The source of AE may arise from multiple phenomena depending on the type of material. Most sources of acoustic emissions are related to damage [28]. The detection of these emissions is commonly used for predictions of material defects. The disadvantage is that AE depends on the used load [29]. This means that come disconnections may not generate a detectable acoustic emission.

Unlike most other approaches to non-destructive testing, the AE method detects only active defects occurring inside the monitored structure. These defects may occur only during stress (mechanical, temperature or chemical stress) within the monitored structure. Passive defects or the shape of the structure do not significantly affect the localization of the AE [30].

The acoustic emission occurs in the AE source upon the release of energy due to stimulation by internal or external tension. The AE event is emitted through irreversible dislocation and degradation processes in the microstructure and macrostructure of the material. The released energy is transformed into a mechanical stress impulse spreading through the material as a longitudinal or transverse wave. As soon as the wave hits the surface of the material, it is detected by a piezoelectric sensor. The signal detected by the AE sensor and transposed to an electric form is referred to as an AE signal [31].

The specific acoustic emission sources may be used for deducing the initiation and distribution of cracks [32,33]. Much effort is devoted to the study of the precise identification of the sources of acoustic emissions, and many algorithms were designed to define the sources of acoustic emissions. These algorithms could be divided into two groups. The first is a non-iterative group (these algorithms assume the same velocity for all stations, thus being relatively inflexible in dealing with variable velocity models). The second group is iterative algorithms (the derivative method, the sequential search method, the genetic algorithm and the simplex method) [34,35]. These algorithms are based on the assumption that the acoustic waves spread directly from their source along a line to the sensor. Thus, they assume the environment in which the waves spread is sufficiently homogenous for generating acoustic waves. Such types of algorithms lose their precision in the case of two different environments or the case of refraction of the generated waves [35].

The localization of the defects was analyzed during the stress of tested specimens using three-point bending. Two acoustic emission sensors of type IDK09 (Dakel company, Prague, Czech Republic) were placed on the testing specimens (see Figure 9b). Subsequently, the tested specimen was subjected to stress by bending using the Testometric M350-20CT electro-hydraulic device (see Figure 8 and Figure 9). The static load application was synchronized with the acoustic emission, meaning both devices were turned on simultaneously. According to technical standard EN 310, the bending stress was applied with the rate of 3 mm/min of moving the transverse load.

The data from the electro-hydraulic device connected to a PC were exported, providing dependencies time/strength, bend/strength. The acoustic emission activity was monitored using the DAKEL-ZEDO system [36]. A two-channel ZEDO-AE unit intended for processing acoustic emission signals was used for the actual measuring. The monitoring of the acoustic emission activity took place along with establishing the bending properties. Thus, the identical specimens were used. The attachment during the three-point load bending test and location of the AE sensors is apparent in Figure 9.

Subsequently, the dependencies of the individual analyzed properties enabled the localization of the formation of defects upon static stress of the cement-bonded particleboards using flexural bending load. The total increase of the signal was 64 dB (34 dB pre-repeater + 30 dB software amplification on the measuring card), the threshold value for the individual hits was 31.482 µV (30.0 dBAE), the sampling of the hits was set to 10 MHz.

**Figure 10:**
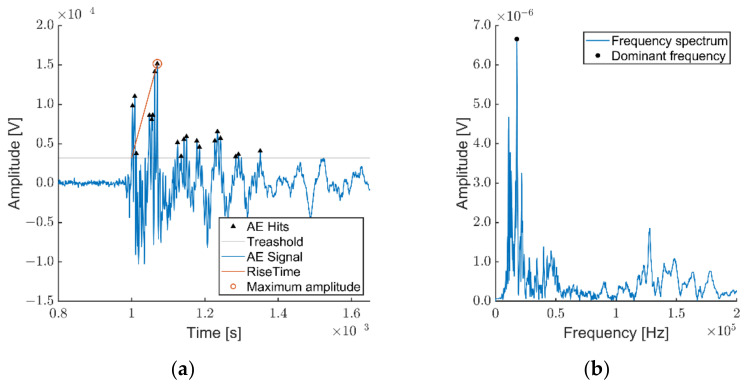
The depiction of the actual AE signal: (**a**) signal of the length of 32,824 samples, recorded during the sample frequency of 10 MHz, 20 present AE hits, rise time equal to 70 µs, length of emission equal to 350.ms, maximum amplitude equal to 145 µV; (**b**) depicted frequency spectrum with a marked dominant frequency equal to 17.6 kHz.

Acoustic emission signals from both sensors used were recorded over the course of the measuring. An example of one individual signal is shown in previous graphs (see Figure 10), indicating the main parameters evaluated within this publication. Almost twenty different parameters are typically recorded according to standardized procedures [37].

In evaluating the simple test of bending, bending strength and elasticity modulus are among the main obtained parameters. The acoustic emission documents the increasing branch of the working diagram up to the moment of damage and the subsequent decreasing branch. Each individual new defect created by the effect of the load strength is documented through a recorded signal. To interpret the output measurements, the Kaiser effect [31], designed in 2007 by Grosse and Ohtsu, may be used for the purpose of monitoring cyclically stressed reinforced iron-and-concrete elements by AE. Two ratios may be used for this interpretation that can be expressed as follows:

RA = rise time/max amplitude,(4)

Average frequency (F_a_) = Number of AE hits/duration (5)

**Figure 11:**
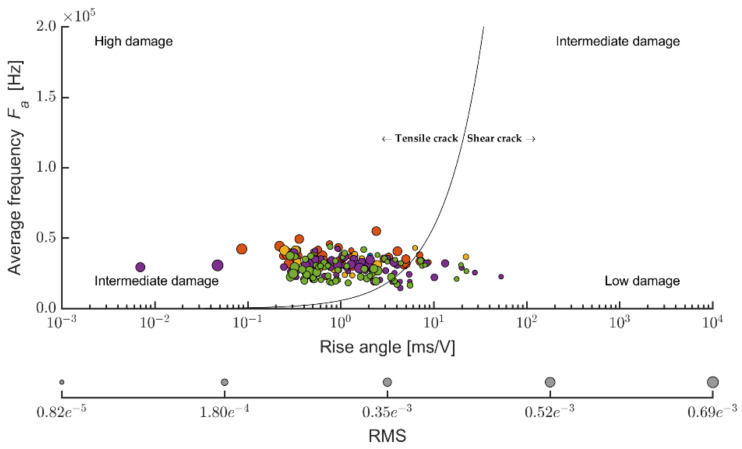
Scheme of AE evaluation interpretation using the calm ratio expressed by the average frequency and load ratio expressed using the RA value.

The Kaiser effect documents the accumulation of damage in the evaluated construction upon cyclical stress. It is thus closely related to the mechanical integrity of the evaluated construction [53,54,55,56,57]. When we express the measurements of acoustic emissions using the RA and F_a_ values, the result is the dependency depicted in Figure 11. Thus, should we apply stress upon an element that is lightly built without damage (caused by cyclical stress), the individual emissions will fall into the left-bottom and right-upper area of the diagram. On the contrary, should the emission show high values of *F_a_* and relatively low values of the stress ratio RA, these are significant defects that Ohtsu et al. relate primarily to tensile defects. On the other hand, defects falling below the shown limit line left bottom are mostly assigned as friction-type damage, thus closer to fatigue defects.

The line dividing the area into tensile and friction defects is set up experimentally (in the laboratory), and publications state various ratios from 1:7000 to 1:100,000 [56,57]. The value in the standard differed for various materials and must be established experimentally [58]. In this case, the value of 1:100,000 *A_f_:RA* was set upon the evaluation of all thus-far executed measurements. The x-axis is expressed in a logarithmic scale for clarity. The size of the individual points mutually differentiates by the root mean square (RMS) of the given signal that could be seen as the weight of the emission.

### 3.5. Microstructural Analysis

A Keyence VHX-950F optical microscope (Keyence, Japan) was used to supplement outputs of acoustic emission. This enabled focusing on areas where defects were identified in higher amounts or energy. Localities where the increased occurrence of defects was identified, however, where the final damage of the test specimen did not occur, were primarily emphasized. Furthermore, cracks in the area of the definitive damage of the test specimen were analyzed.

## 4. Results and Discussion

### 4.1. Physical and Mechanical Parameters

**Figure 12:**
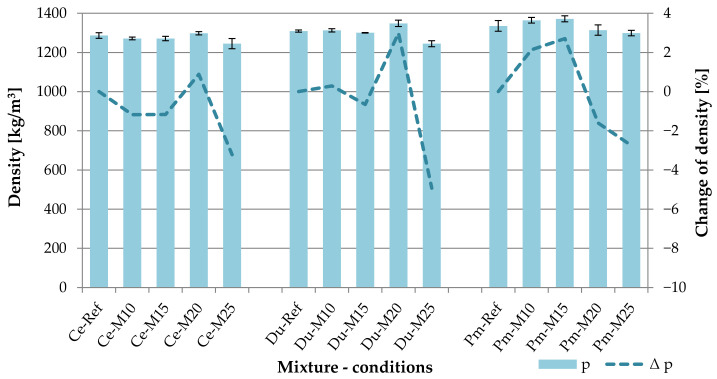
Comparison of density of tested boards; columns—density (p), curves—change of density (Δp).

A Density is one of the monitored and evaluated parameters of cement-bonded particleboards. According to EN 634-2, the minimal required density value is 1000 kg/m^3^. The achieved results, including their graphic depiction, are shown in the following table (see Table 3) and graph (see Figure 12). It is apparent that not all tested types of boards achieve density >1000 kg/m^3^ even after 250 frost/defrost cycles.

The above density values fail to enable the recording of a clear dependency of the mixture compositions and the conditions of their adverse exposure. A more notable decrease in density, approximately 3–5%, is notable only in the case of a higher number of frost/defrost cycles (specimen sets M20). An interesting finding is a fact that in the case of some sets, an increase in density. However, this increase ranged under 3%. With respect to the reached density values, these are rather negligent differences. No clear dependencies could also be concluded through the evaluation of absolute errors (graph in Figure 12 shown as error line segments) of densities established for each tested set. The absolute errors reach values in the interval from 1.5 kg/m^3^ to 27 kg/m^3^, which corresponds to the relative error range of 0.12% to 2.02%. Very similar density values without apparent dependency on the composition of the tested boards types could be attributed to the lower dose of substitution components. Additionally, the high compatibility of the alternative substituents DU and PM with the cement-bonded boards plays a role.

**Figure 13:**
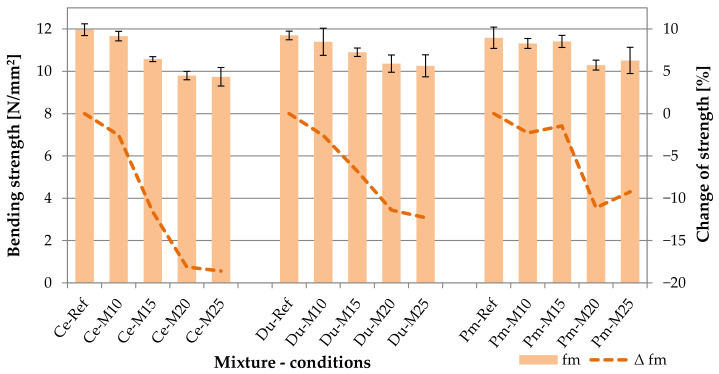
Comparison of bending strength; columns—bending strength (f_m_), curves—change of bending strength (Δf_m_).

Bending strength is one of the most important usage properties in the case of cement-bonded particleboards. The achieved results of this strength, including their graphic depiction, are shown in the following table (see Table 4) and graph (see Figure 13). According to EN 634-2, the minimal required value of bending strength is 9 N/mm^2^. All tested cement-bonded particleboards reach the average bending strength >9 N/mm^2^ and this is also after 250 frost/defrost cycles. This finding is very useful from the perspective of modified boards which thus proved very good durability.

The results of all tested types of cement-bonded particleboard prove the negative effect of adverse environment on bending strength. The ratio of strength before and after the stipulated number of frost/defrost cycles *R* (according to EN 1328) ranges between 0.98 and 0.81 (which corresponds to the decrease of bending strength from 2.3 to 18.6%).

Boards Ce-Ref reached the highest strength (without exposure to frost conditions), while the difference in comparison with modified boards Du-Ref and Pm-Ref are not very significant (0.3 to 0.4 N/mm^2^). The decrease in strength, due to the effect of a lower dose of cement in the boards, corresponds with outputs presented by Zhou et al. [21]. Zhou focused on the possibility of using waste wood treated with chromate copper arsenate (CCA) in particleboards. Boards with varied ratios of wood and cement were tested, specifically from 1.0 to 4.0 (cement/wood). In the area of cement/wood ratio = 2.0 to 3.0, bending strength ranged from 8.44 to 9.52 N/mm^2^.

The modified boards Du and Pm show a lower decrease in bending strength due to an adverse environment, as apparent from 150 frost/defrost cycles. This finding is particularly interesting because in both cases, the cement was replaced with inert filler. However, in the case of the Pm boards, this fact could be attributed to the substitution of 4% of spruce chips with an alternative material. Aside from the chips, PM also contains the cement matrix that is also present on the chips. Additionally, the chips contained in the PM are already once mineralized (both by ions of sodium silicate and lime ions from cement). In the case of the Du boards, the lower decrease in strength due to the frost effect could be attributed to the alternative component used.

The DU alternative raw material contains many more fine components compared to PM. DU is also an inert filler, but thanks to the particle size, there is a better bonding and mutual effect with the matrix in the cement-bonded particleboards. The different ratio of cement and wood chips could also have a beneficial effect on frost resistance, which is in part supported by the results of a study conducted by Soroushian et al. [13]

Among other findings, he concluded that the ratio of cement and wood matter positively affects the frost resistance of cement-bonded particleboards. Specifically, Soroushian found that in the case of the wood/cement ratio = 0.28, higher bending strengths were achieved upon exposure to 25 frost/defrost cycles, compared to the wood/cement ratio = 0.35. However, it must be taken into account that the course of the frost/defrost cycles presented in [13] was different from the course of cycles described in Chapter 3.2. According to [13], the cycle lasted a total of 24 h, where 12 h were freezing and 12 h thawing.

However, absolute errors and relative errors (diversions from averages) are also essential from the perspective of the achieved bending strength values. Boards with modified composition are characterized by a higher degree of results variability. This finding refers to values established upon exposure to frost/defrost cycles. The Pm boards show the most variable results. Thus, in this respect, a certain relation between the modification of the board composition with an alternative material (inert filler) at the expense of primary composites with a bending strength may be observed. Results presented by Wang et al. [8] also confirm the effect of the identified variability of the bending strength of PM (in comparison with Ce).

According to Wang et al., the decreased dose of cement at the expense of the alternative filler (construction waste wood) causes the increase of the divergence from the average value of bending strength. However, when comparing the absolute error with the findings of Fuwape et al. [14], it may be concluded that the Du and Pm mixtures are overall characterized by very low variability of the individual measurement. Fuwape et al. found that the cement-bonded particleboards density of 1175 kg/m^3^ achieved a bending strength of 8.11 N/mm^2^ with an absolute error of 2.04 N/mm^2^ (approx. 25.2%).

However, Fuwape et al. analyzed properties with the content of the waste-paper and waste-wood filings. In this respect, the alternative components DU and PM could be seen as more compatible and thus better mutually reactive with the cement-bonded particleboard matrix.

**Figure 14:**
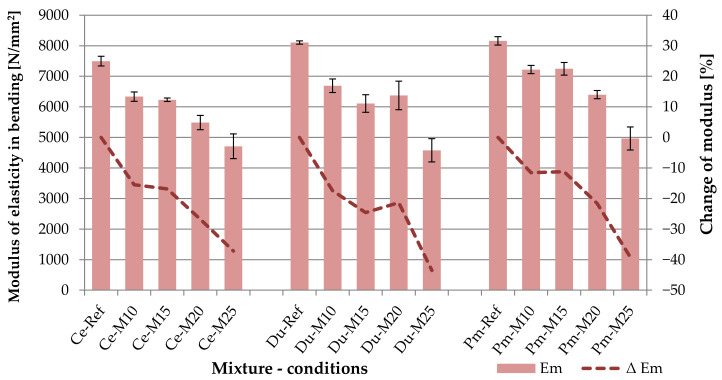
Comparison of modulus of elasticity in bending; columns—modulus of elasticity in bending (E_m_), curves—change of modulus of elasticity in bending (ΔE_m_).

By stressing the boards by bending, it was possible to identify both bending strength and the modulus of elasticity in bending. This property is essential from the perspective of evaluating the behavior of boards with modified formulae under load. The achieved results are shown in the following table (see Table 5) and graph (see Figure 14). According to EN 634-2, the minimum required value for the modulus of elasticity in bending is 4500 N/mm^2^ (Class 1), 4000 N/mm^2^ (Class 2), respectively. All tested cement-bonded particleboards reach average values of the modulus of elasticity in bending >4500 N/mm^2,^ and this is also after 250 frost/defrost cycles.

The decreasing trend in the modulus of elasticity in bending in the individual formulae, due to the effect of the frost/defrost cycles, approximately copies bending strength. Nonetheless, some fundamental differences may be observed here in comparisons of the modulus of elasticity, specifically in boards Ce-Ref, Du-Ref and Pm-Ref. The boards with modified compositions, meaning Du-Ref and Pm-Ref, achieve higher levels in modulus of elasticity in bending than in the case of the reference Ce-Ref boards. The only exception are values of the modulus of elasticity in bending established after the exposure to 250 frost/defrost cycles, meaning Du-M25 and Pm-M25.

Fuwape et al. [14] also reports the different course of strength and the modulus of flexibility in bending of cement-bonded particleboards with modified composition. The contradictory trend in strength and modulus of elasticity in bending is also confirmed in the results presented by Savastano et al. [19]. In this study, Savastano focuses on cement and pine chip boards. The pine chips are substituted by up to 12% by kraft pulps from sisal and banana waste and from Eucalyptus grandis pulp mill residues. However, an essential fact is that the above studies do not cover the effect of frost/defrost cycles on the board properties. The authors [14,19] only analyzed the effect of the modification on the composition of cement-bonded particleboards and this, specifically, by substitution of wood chips.

Upon closer examination of the obtained values (see Table 5), specifically the bend in the elastic area (meaning between 10 and 40% of maximum strength) in comparison with the bending strength, it was found that the Du and Pm boards are less flexible. This refers to boards that are not exposed to the adverse conditions of frost, meaning Du-Ref and Pm-Ref. However, these differences are not very significant in comparison with the reference Ce-Ref boards. The bend (deformation) of all types of boards upon reaching the bending strength is similar. This fact is related to the modification of the composition of chips. The primary chips were replaced by chips from alternative materials (DU—1% and PM—4%). The spruce chips contained in the DU and PM are already mineralized and of much smaller size in the case of DU.

Additionally, the modification of the matrix of the boards must be considered (DU—6% and PM—4%). However, the behavior from the perspective of elasticity of the modified boards upon reaching 250 frost/defrost cycles is different. Du-M25 boards particularly show the greatest decrease in the modulus of elasticity. Another interesting finding is that while the modulus of elasticity drops more notably in all board types after 100 frost/defrost cycles, after 150 cycles, it does not appear to be significant (compared to the 100 cycles). This phenomenon was observed in all three board types: Ce, Du, as well as Pm. As in the case of bending strength, the relative errors increased with the increasing number of frost/defrost cycles.

However, upon comparing the relative errors of bending strength and the modulus of elasticity in bending, it is apparent that the individual values of the modulus show larger variances. The comparison of relative errors only confirmed the increased variability of results in the case of Du boards, compared to Ce. Thus, it cannot be clearly stated that the modification of the compositions of boards with alternative materials would have any effect on the increased variability of the individual values of the modulus of elasticity in bending (the comparison of Ce and Pm).

The ratio of bending strength to the modulus of elasticity in bending (f_m_/E_m_) approximately corresponds with the results presented by Okino et al. [20]. This is an interesting finding, as it refers to a fundamentally different composition of the cement-bonded particleboards in comparison with the study [20] and the research presented here.

**Figure 15:**
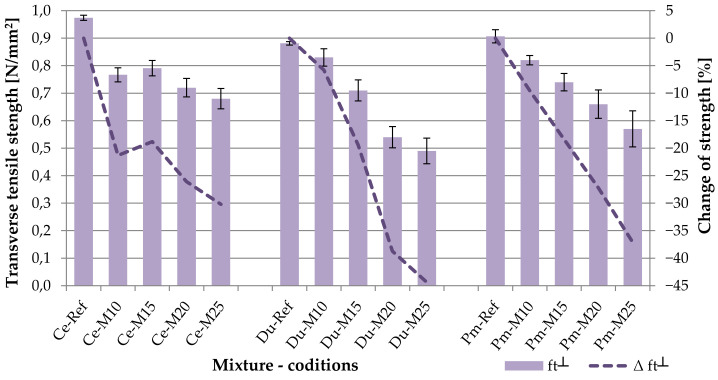
Comparison of transverse tensile strength perpendicular to the plane of the board; columns—transverse tensile strength (f_t┴_), curves—change of transverse tensile strength (Δ f_t┴_).

The transverse tensile strength perpendicular to the plane of the board characterizes the cohesiveness of the cement-bonded particleboard in the direction of its pressing process. This characteristic is significant for the boards, as most particles are oriented along the plane of the board during the densification. Thus, the cement-bonded particleboard shows different properties in the transverse direction, including strength. The results of establishing the tensile strength perpendicular to the plane of the board are shown in the following table (see Table 6) graph (see Figure 15). According to EN 634-2, the minimum required tensile strength perpendicular to the plane of the board is 0.5 N/mm^2^. All tested cement-bonded particleboards reach average tensile strength ≥ 0.5 N/mm^2^, and this even upon 250 frost/defrost cycles.

The results of testing tensile strength perpendicular to the plane of the board confirm the necessity of establishing all essential properties of the given material, as the frost/defrost effect was most notable here. The Ce boards, meaning the reference mixture, are shown as the best in the evaluation. However, the course of the curves characterizing the percentage in a decrease of tensile strength is interesting (see Figure 15—dashed curves). In the case of Ce, a sharp decrease may be observed upon the effect of as little as 100 frost/defrost cycles. The DU and PM alternative raw materials do show a sharp but rather gradual decrease of tensile strength due to the effects of the adverse environment. The most abrupt decrease of tensile strength, perpendicular to the plane of the board, is shown in the case of boards made from the Du-M25 materials, specifically 44%. The requirement of the relevant technical norm [48] is at least 0.5 N/mm^2^. Thus, it is evident that, upon reaching 250 frost/defrost cycles, tensile strength is clearly very significantly affected. This phenomenon is most likely related to the fact that in the case of the Du boards, a larger amount of cement was replaced by the alternative component, specifically by 7% of the dust from board processing (where DU reaches approx. 25% of the wood matter). The relative error (divergence from the average value) increases with the number of frost/defrost cycles. This phenomenon is most notable in the case of DU and PM alternative raw materials (see Table 6).

As is apparent from the comparison of achieved average values of tensile strength perpendicular to the plane of the board with the results presented by Okino et al. [20], the Du and Pm boards achieved notably higher values (and this even upon 250 frost/defrost cycles). The research presented by Okino et al. shows the results of this characteristic (IB—internal bond) within the range of 0.19 to 0.40 N/mm^2^.

### 4.2. Acoustic Emission

The courses of the value of several characteristics were monitored and evaluated through the analysis of the course of mechanical bending load, specifically the combination of traction and bending, using acoustic emission. The primary focus was the identification and localization of defects forming during the gradual application of load on the cement-bonded particleboards. For this purpose, so-called ‘hits’ were scanned and recorded. This parameter characterizes events that occur due to the damage of the material, thus enabling the recording and localization of active defects. The graphs below show the courses of the values of representative specimens. The first set of graphs depict the dependency of the cumulative curves of the hits (from the entire monitored area of the test specimen) in bending and tension (see Figures 17, 20 and 23). A similar type of interpretation of measuring a three-point bending along with occurring defects, using acoustic emission in diagnostic practice, is presented, for example, by Sagar et al. [58] However, Sagar focuses on concrete, not cement-bonded particleboards. The course and a cumulative number of overshoots are expressed here in relation to the deflection of the loaded specimen. To be able to evaluate the course of load on the cement-bonded particleboards in the bend in closer detail from the perspective of formation and localization of defects, the hits were transposed into three-dimensional graphs (see Figures 17, 20 and 23). The last graph sets are the rations of the average frequency and RA value (see Figures 18, 21 and 24), which, according to [57,58], enables the evaluation of the ratio of tensile and friction tensions occurring in the tested specimen upon bending flexural load (according to [51]). The technical regulation [51] shows an imprecision, particularly in establishing the elasticity modulus in bending, as this characteristic may also be affected by the occurrence of friction tension.

**Figure 16:**
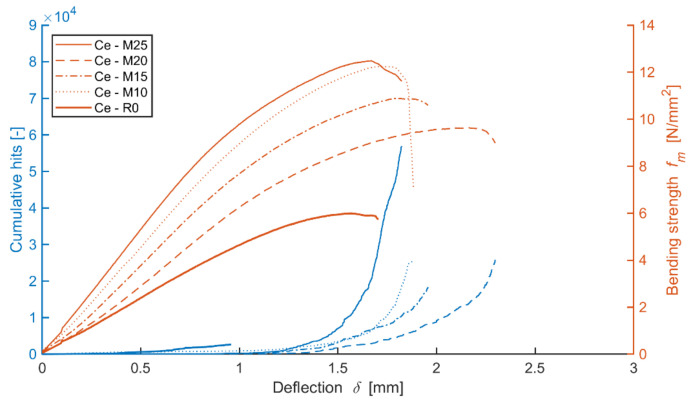
Comparison of bending strength, deflection and cumulative hits of representative test specimens (during mechanical load) of mixture Ce (reference boards) before and after frost/defrost cycles.

The reference boards gradually show a decrease in bending strength with the increasing number of frost/defrost cycles. The record of the acoustic emission activity (see Figure 16) shows that the lowest number of defects occur in the boards exposed to 250 frost/defrost cycles. On the contrary, the reference specimen, meaning one without exposure to an adverse environment, shows a rather steep increase in acoustic emission upon the bend of approximately 1.3 mm, as well as a higher number of forming defects. This fact indicated the presence of defects in frost-stressed cement-bonded particleboards even prior to its bending load. Such defects could be seen as passive, meaning they were present in the boards prior to exerting the load. Thus, such passive defects were not recorded by the AE. Additionally, there is a damping of the hits (mechanical waves) by the already existing defects and defects in the structure caused by the frost/defrost cycles. The graph (see Figure 16) shows how the inclination of the curves characterizing the dependence of bending strength on the deflection is changing. This indicates the changes in the elasticity modulus in bending and approximately corresponds to the results of the physical and mechanical parameters (see Table 5 and Figure 14). The elasticity modulus decreases with the increasing number of frost/defrost cycles. The only exception, in this case, is specimen M25, after 250 cycles of adverse exposure. The structure of this specimen was already so damaged by the frost/defrost cycles that it was definitively damaged upon a relatively low bending and tension.

**Figure 17:**
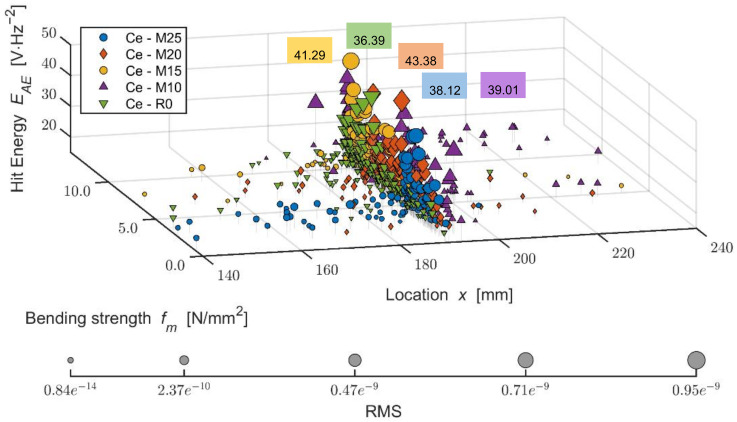
Comparison of bending strength and defects development (hit energy with maximum; larger points = higher RMS) during static mechanical load in monitored area of test specimens; mixture Ce (reference boards) before and after frost/defrost cycles. x = 190 mm—middle of the specimen.

The graph (see Figure 16) characterizes the number of active defect events occurring during the bending load of the tested specimen in relation to its deflection and tension, regardless of the location of the defect. The energy was evaluated for the locations of active defects and a more detailed analysis of the behavior of the cement-bonded particleboards (higher energy suggests a higher degree of damage of the material at the given moment) in the individual AE events (hits). The following graph (see Figure 17) depicts the location of the individual AE events in relation to their energy and bending strength. It is very interesting to observe the development of these parameters and how they change with increasing tension until the point of complete damage to the test specimen. From the beginning, the formation of the defects is primarily apparent in the area of the cylindrical stress-test head of the press (meaning in the center of the tested specimen). Later it is evident that not many defects are formed in the flexible area (characterized by the interval of 10% and 40% if at maximum load strength) and if so, they are characterized by lower energy.

Upon reaching the maximum bending strength of the board in bending, defects are formed even further away from the cylindrical stress-test load head. However, these defects are characterized by lower energy. The finding that a test specimen after 100 cycles shows higher-energy defects already at a rather low tension in the flexible area is also interesting. It is apparent that with the increasing number of frost/defrost cycles, the concentration area of occurring defects moves toward the flexible area of the load. It is further evident that the distribution of defects decreases from the center on, meaning that they are mainly concentrated in the center area of the test specimen. For example, upon applying the load to the Ce-M25 board, a higher-energy defect was recorded at the bending tension as low as approximately 1 N/mm^2^.

The defects on the Ce-M20 board occurred at lower tension, meaning up to 1 N/mm^2^, having negligible energy, including those located further away from the cylindrical load head of the press.

**Figure 18:**
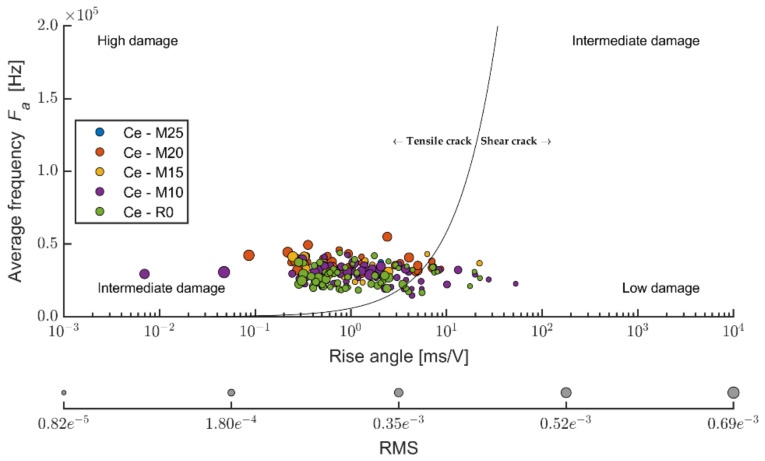
Comparison of average frequency and RA values—relation between tensile and shear cracks in monitored area of test specimens; mixture Ce (reference boards) before and after frost/defrost cycles.

The ratio between applying tension load (bending) and friction, according to [51], the Ce mixture (see Figure 18), points to the fact that the friction component of the load upon the stipulated elasticity modulus in bending represents a considerable part. According to the values of the ratio of the average frequency and RA values, it is evident that boards exposed to 100 cycles were also considerably stressed by friction tension. Upon a higher number of frost/defrost cycles (meaning from 200 on), the cement-bonded particleboards were mostly more stressed only by tensile pressure (in bending). The cause could be an increasing number of defects in the structure due to the effects of frost. These defects appear, among other aspects, through decreased cohesion in the cement matrix and wood chips. The chips may also become more brittle due to the effects of an adverse environment. As a result of these structural changes of the boards (in the area of the final damage), the chips are pulled out of the matrix or damaged by traction (perpendicularly to the direction of the load). Thus, only tensile stress is transferred in the loaded area of the test specimen. Due to the character of changes in the structure, the structure is thus unable to transfer friction tension. It may be concluded that according to the calculation formula of the elasticity modulus in bending, according to [51], the lesser indicative values of the modulus regard the boards exposed to 100 frost/defrost cycles. In case of this set of test specimens, more damage occurs in the area of friction (see Figure 17).

**Figure 19:**
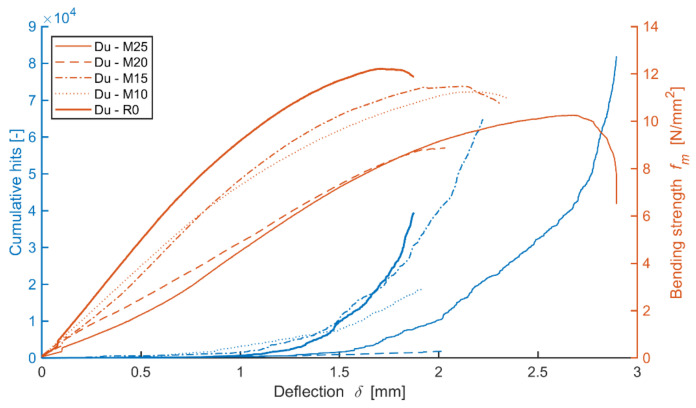
Comparison of bending strength, deflection and cumulative hits of representative test specimens (during mechanical load) of mixture DU (boards modified by dust DU) before and after frost/defrost cycles.

The boards modified by dust from cutting, DU, perform during bending loads somewhat differently (see Figure 19) from the reference mixture Ce boards. The decrease in strength and elasticity modulus in bending due to the increasing number of frost/defrost cycles is similar. However, a more significant decrease in modulus values along with an increase in defects forming (meaning active) was recorded during the stress tests due to the effects of frost. Nonetheless, it was not possible to observe a clear dependence between cumulative curves of the hits and the course of the tension/deformation curves.

**Figure 20:**
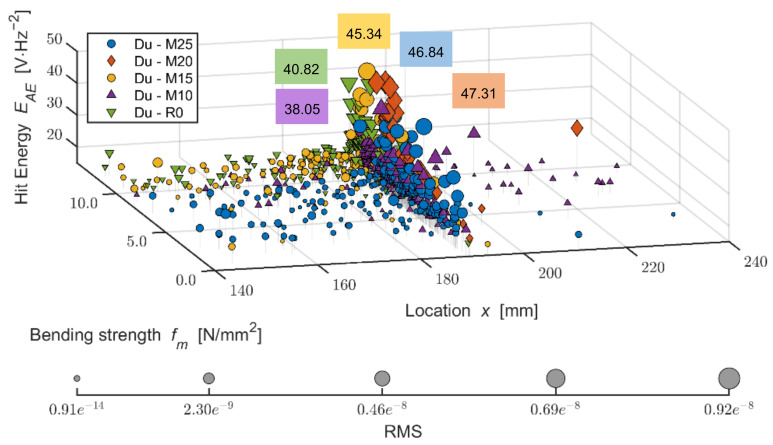
Comparison of bending strength and defect development (hit energy with maximum; larger points = higher RMS) during static mechanical load in the monitored area of the test specimens; mixture DU (boards modified by dust DU) before and after frost/defrost cycles. x = 190 mm—middle of the specimen.

The Du board without any effect of adverse environment shows a rather steep increase of active defects during the load within 1.4 mm of the area of bend, where the pressure reaches maximum limits, meaning damage. With the increasing number of frost/defrost cycles, this steeper increase is notable in the case of higher deflection, for example, from approximately 1.5 mm (Du-M15) or 2.0 mm (Du-M25). A more detailed analysis of the performance of the boards during their bending load from the perspective of defects detected by AE is shown in the three-dimensional graph below (see Figure 20). As is apparent from the graph, the Du boards show a larger number of defects occurring over the course of the load. It is also apparent that the energy of hits reaches higher values than in the case of the Ce boards. The defects are not as concentrated in the area of the load applied by the head of the press. The frost-stressed specimens also show defects in the flexible area.

The varied distribution of the defects in test specimens M20 and M25 (meaning those exposed to 200 and 250 frost/defrost cycles) is interesting. The M20 board shows the increased energy of hits in a linear manner, where the defects were detected using the AE at as low tension as approximately 1 N/mm^2^. On the contrary, test specimens exposed to 250 cycles, the M25, could be characterized by a more gradual increase in the number and energy of hits. The defects with lower hit energy are distributed across the area of approximately ±50 mm from the load from the head of the press. This distribution increases and reaches its maximum at the moment of damage to the M25 boards.

**Figure 21:**
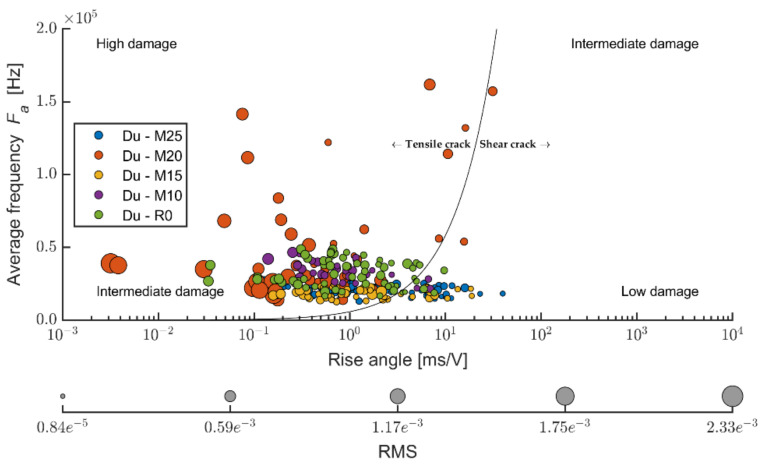
Comparison of average frequency and RA values—relation between tensile and shear cracks in the monitored area of test specimens; mixture DU (boards modified by dust DU) before and after frost/defrost cycles.

The evaluation of the ratio of the average frequency or RA values (see Figure 21) enables the finding that the DU boards are stressed by the traction to a slightly larger degree than Ce boards. However, it is not possible to observe clear dependencies of the ratio traction/friction ratio to the number of frost/defrost cycles. According to the values of the ratio of the average frequency to the RA values, it is evident that boards stressed by 150 cycles and 250 cycles were also affected by friction tension. At 100 and 200 frost/defrost cycles, the cement-bonded particleboards were affected practically only in tensile load (in bending). The effect of the adverse environment was, in this case, showing differently from the Ce boards.

However, a definitive and objective conclusion cannot be deduced within this scope of executed experiments to clarify this phenomenon (the effect of frost) with its logical justification. Furthermore, the available relevant professional publications do not provide data from testing cement-bonded particleboards corresponding with the above finding.

**Figure 22:**
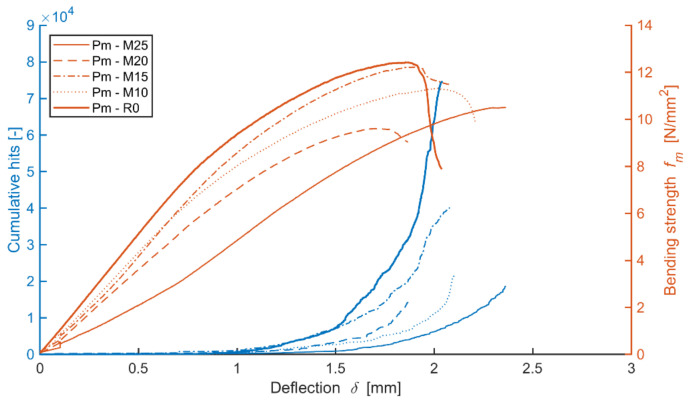
Comparison of bending strength, deflection and cumulative hits of representative test specimens (during mechanical load) of mixture PM (boards modified by particle mixture PM) before and after frost/defrost cycles.

Despite similar values of bending strength, the Pm boards show a fundamentally different course of cumulative curves of hits (see Figure 22) than boards Ce and Du. In this case, active defects form in a completely different manner. The steepest increase in the number of defects was identified in the case of reference-value boards. With the increasing number of frost/defrost cycles, there appeared a gradual decrease in the number of defects. With respect to the bending strength achieved, it is, therefore, possible to assume the formation of a larger percentage of defects occurring prior to the test during the frost/defrost cycles.

With the rising number of frost/defrost cycles, it is possible to note a more gradual increase of cumulative hits. Gradually, there is a decrease in the number of active defects due to the effects of frost. This phenomenon indicates that the boards exposed to frost have already shown defects (meaning passive defects) prior to exposure to bending load. Strength, as well as the elasticity modulus in bending, decreased due to the effects of adverse environments. For example, the difference in the course of formation (cumulating) of hits in the case of the Pm boards exposed to 100 and 150 frost/defrost cycles is interesting. Both the strength and elasticity modulus of these sets, meaning M10 and M15, are very similar. However, the course of the cumulative curves of AE events is rather different. This fact highlights the different behavior of these sets of boards over the course of bending load. On the contrary, the cumulative curves of hits confirmed, and to a certain degree clarified, the higher the bending strength in boards exposed to 250 cycles, as opposed to boards exposed to 200 frost/defrost cycles. For some reason, the M20 boards were affected by the frost more than the M25 boards—imperfections in the structure such as from production, non-hydrated cement that gradually hydrated, possibly the damage of grains of non-hydrated cement and its hydrations, etc.

**Figure 23:**
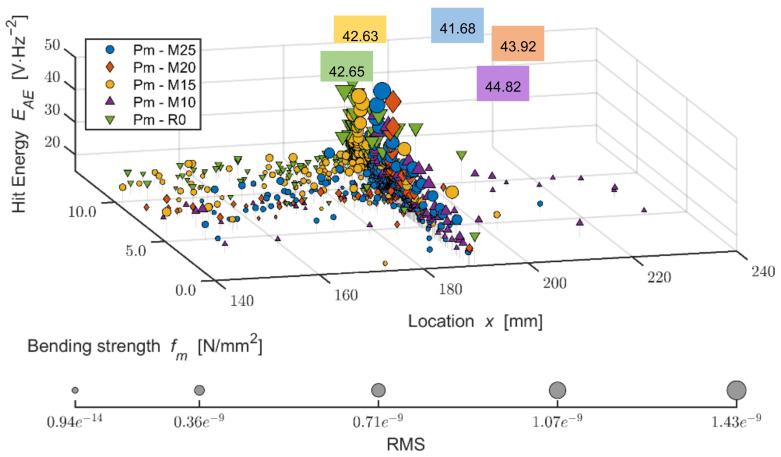
Comparison of bending strength and defects development (hit energy with maximum; larger points = higher RMS) during static mechanical load in the monitored area of test specimens; mixture PM (boards modified by particle mixture PM) before and after frost/defrost cycles: x = 190 mm—middle of the specimen.

The spatial depiction of the distribution of energies of forming defects in relation to bending load in the case of the Pm boards (see Figure 23) proves a different behavior from both the Ce and Du boards. The even energies (hits) are similar, as in the case of the Du boards (see Figure 20). However, the localized events are concentrated closer to the area of damage, meaning that reaching bending strength (thus damaging the test specimen). It is of interest that the specimens exposed to 150 frost/defrost cycles show higher energy of hits. This finding is true for both Pm boards and the reference Ce boards. The defects formed close to the area of damage show lower energy, which is an essential difference in comparison with the Du boards. Additionally, the finding that fewer defects form in the flexible area of the Pm boards is important, possibly with defects with lower energy than in the case of Du and Ce boards. This finding could be related to the elasticity modulus values (see Figure 14), where the Pm boards show the highest values in comparison with Ce and Du boards.

**Figure 24:**
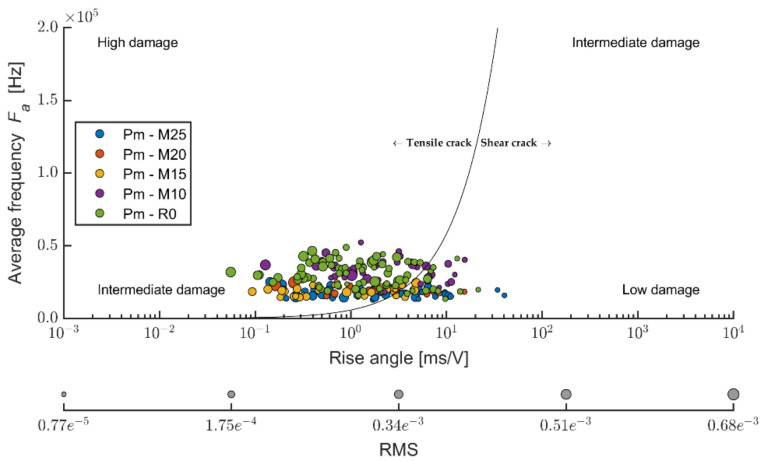
Comparison of average frequency and RA values—relation between tensile and shear cracks in the monitored area of test specimens; mixture PM (boards modified by particle mixture PM) before and after frost/defrost cycles.

Dividing the traction and friction area in the case of the Pm boards (see Figure 24) approximately corresponds with the results of the Du boards. The ratio of traction and friction cracks is thus more favorable than in the case of the reference boards (from the perspective of evaluating the elasticity modulus in bending according to [51]). A certain percentage of points (cracks) fall into the friction area. However, that is not very significant. Thus, in this case, the friction component does not significantly contribute to the effect on the achieved results of the elasticity modulus.

It can be concluded that the established values of elasticity modulus in bending (specifically, traction in bending) of the Du boards (in comparison with Ce) are more evidential. The friction defects on the Du boards were not identified to such a degree as in the case of the Ce boards. The values in the graph incline toward an approximate dependency of the number of frost/defrost cycles to the size of the friction component, specifically of defects formed during friction. With the increasing number of frost/defrost cycles, the ratio friction/traction thus gradually changes toward the traction stress and thus also defects.

In comparison to the Ce and Du boards, the Pm boards, upon reaching 150 frost/defrost cycles, show an interesting phenomenon: three areas of tension defects were identified here over the course of bending load. These areas are considerably distant from the edge of friction and traction defects toward the traction area.

Additionally, other authors, for example, Jiang et al. [59], report an approximately similar finding of a ratio of traction and friction defects in the case of reference boards (without exposure to the conditions of frost/defrost cycles); see Figures 18, 21 and 24. As is apparent from results published by Jiang, the evaluation of the destructive test from the perspective of Af:RA on laboratory specimens enables the determination of which type of damage prevails in AE. Therefore, it is possible to determine whether this is more of fatigue friction damage or fragile jump damage in the case of the reference non-degraded specimens. However, Jiang et al. focused on fundamentally different materials than cement-bonded particleboards.

### 4.3. Microstructure

**Figure 25:**
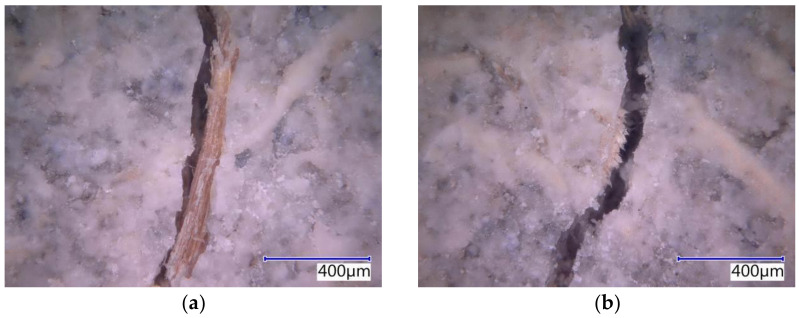
Detailed structure of crack in Ce-Ref—pictures from optical microscope: (**a**) chip oriented along with the crack; (**b**) defects both in matrix and chips.

**Figure 26:**
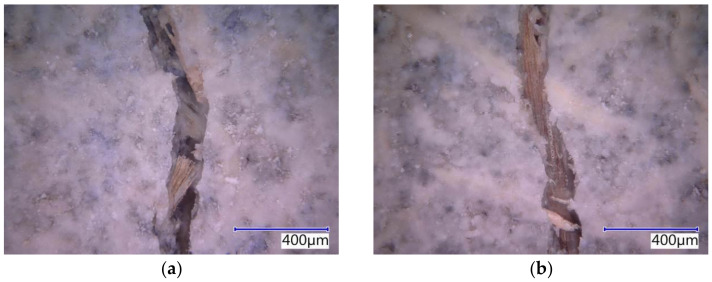
Detailed structure of crack Ce-M20—pictures from optical microscope: (**a**) chip pulled out from the matrix; (**b**) chip oriented along with the crack pulled out from the matrix.

**Figure 27:**
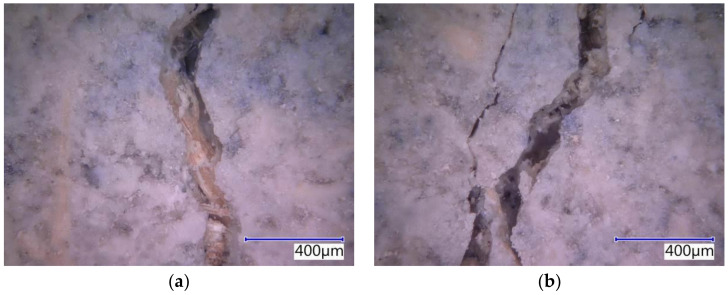
The detailed structure of crack in Du-M20—pictures from optical microscope: (**a**) deteriorated chip partially pulled out from the matrix; (**b**) deteriorated locality with probable cluster of dust DU.

**Figure 28:**
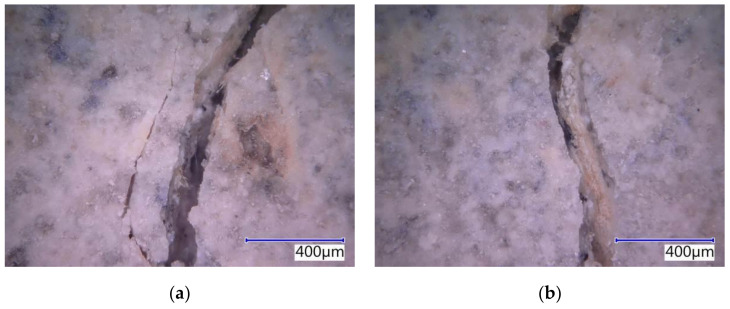
Detailed structure of crack PM-M20—pictures from optical microscope: (**a**) dominant crack with minor crack and defect of matrix and chips; (**b**) deteriorated chip oriented along the crack.

**Figure 29:**
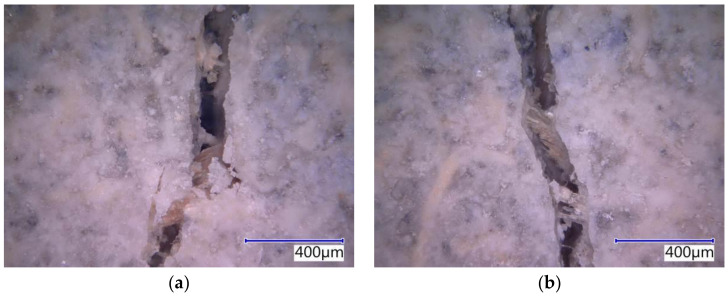
Detailed structure of crack PM-M25—pictures from optical microscope: (**a**) chip pulled out from the matrix; (**b**) multiple defect chips.

The subject cement-bonded particleboards were tested in detail using the Keyence VHX-950F optical microscope. The photographs below show selected segments of the test specimens representing typical defects caused by bending load (see Figure 25, Figure 26, Figure 27, Figure 28 and Figure 29). The microscopic analysis focused on the character of the cracks in the traction area of the test specimens. Specifically, a detailed evaluation of the structure of the crack in the area of the course of the AE signal was made, meaning the longitudinal axis in the center of the width of the test specimen. The identification of possible differences and anomalies in the structure was emphasized. It was found that different types of damage occurred from the perspective of mutual effect of the cement matrix and wood chips.

Some locations showed both more damaged chips and matrix (see Figure 25b), serving as evidence of the synergistic effect of the cement matrix and chips, which was primarily found in the case of the boards with the reference Ce mixture. Generally, both matrix and chips were damaged in boards without exposure to the adverse environment (frost/defrost cycles). Additionally, areas where chips were damaged or partly damaged and pulled out of the matrix, were identified. The chips were oriented along the crack (see Figure 25a, Figure 26b and Figure 28b). No direct relation to the orientation of the chips with the different compositions of the boards or the number of frost/defrost cycles could be found. The orientation and distribution of the chips predominantly relate to the production conditions of cement-bonded particleboards. With the increasing number of frost/defrost cycles, mainly defects showing chips pulled out from the cement matrix were found. In this area, the matrix was impaired while the wood chips were not damaged (see Figure 26 and Figure 29a). The pulling of the chip out of the matrix indicates a defect of ITZ (interfacial transition zone between matrix and wood chip). This could relate to the cyclical changes of the chips caused by fluctuations in temperature and humidity. In the presence of water, wood chips are subject to considerable changes in volume. This phenomenon is apparent despite the mineralization (the stabilization of properties) of the chips. The matrix can be gradually damaged around the chips due to their increased volume, resulting in a partial or complete loss of cohesion of the wood chips and matrix.

The detailed analysis of the structure of the defects in the area of fracture of the test specimen supported specific links. However, on the other hand, it is necessary to realize that this is a local analysis of the surface structure of the test specimen. The traction area also reaches a considerable part of the thickness of the board. It is also significant that the outputs of the AE acoustic analysis clearly evidenced that a considerable number of defects occur outside the location of the dominant crack.

## 5. Conclusions

On the basis of performed experiments and their detailed analysis, it is possible to conclude the following:

- The partial substitution of primary components of cement-bonded particleboards with dust DU or particle mixture PM in up to 8% is feasible and profitable;
- The alternative materials, DU and PM, show a slightly negative effect on the strength characteristics but, on the other hand, have a positive effect on the elasticity modulus in bending;
- The modified particleboards Du and Pm show better frost-resistance in the long-term horizon, meaning one year of age of the boards. A lower decrease in bending strength was recorded with the increased number of frost/defrost cycles in comparison with the control Ce boards;
- The acoustic emission proved different courses of damage, which enabled clarification of the different behavior of the mixtures from the perspective of bending properties;
- The location of active defects was executed during mechanical static bending load;
- The defects were found even in the case of lower tension, specifically in the flexible area (where they normally do not form) and are not notably concentrated in the area of the direct effect of the load;
- In the case of the board with the reference mixture Ce, active defects were concentrated mainly under the load head of the press. On the contrary, the modified mixtures at the tension on the limit of damage showed the broader distribution of defects that were also located further away from the load head of the press;
- Upon damage to the modified mixtures, meaning reaching bending strength, higher hit energy was detected;
- The acoustic emission was also used to analyze defects caused by traction and friction. Traction defects are more prevalent than friction defects. However, friction defects were identified in significant amounts and energy levels;
- The initial setting of the RM:Af ratio was established for a CETRIS type material to 1:100,000;
- The differences between the ratio of friction and traction defects due to bending load are evident in the case of the individual mixtures. The highest ratio of friction defects was found in the modified Pm boards.

## Figures and Tables

**Table 1:** Composition of cement-bonded particleboards–standard and modified mixtures.

Compound	Mixture–Number of Compounds [%]		
	Standard Ce	Modified (by DU) Du	Modified (by PM) Pm
Cement	50	44	46
Spruce chips	18	17	14
Dust (from grinding and cutting cement-bonded particleboards)	0	7	0
Particle mixture (from production line of Cement-bonded particleboards)	0	0	8
Water	30	30	30
Hydration additives	2	2	2

**Table 2:** Chemical composition of cement and alternative raw materials used.

Chemical Compound	Amount of Compound [Mass %]		
	Cement	DU	PM
SiO_2_	19.3	10.6	15.2
Fe_2_O_3_	3.1	3.7	4.3
Al_2_O_3_	5.6	14.8	14.1
CaO	59.8	45.9	42.7
SO_3_	3.27	0.36	0.73
Na_2_O	0.16	0.39	1.26
K_2_O	0.73	0.073	0.084
MgO	1.8	0.16	0.061
MnO	0.061	0.018	0.048
Cl^−^	0.065	<0.01	<0.01
wood content—from TOC	---	25.8	20.3

**Table 3:** Results of density testing of modified cement-bonded particleboards.

Mixture	Density [kg/m^3^]	Average Density [kg/m^3^]	Absolute Error [kg/m^3^]	Relative Error [%]
Ce-Ref	1245–1317	1287	14.4	1.12
Ce-M10	1252–1285	1272	6.8	0.53
Ce-M15	1254–1305	1272	11.1	0.87
Ce-M20	1274–1319	1298	8.7	0.67
Ce-M25	1172–1303	1245	25.7	2.07
Du-Ref	1292–1318	1309	5.5	0.42
Du-M10	1288–1328	1313	8.5	0.65
Du-M15	1297–1305	1301	1.5	0.12
Du-M20	1302–1385	1348	16.3	1.21
Du-M25	1211–1288	1245	15.1	1.21
Pm-Ref	1274–1412	1336	27.0	2.02
Pm-M10	1321–1389	1364	14.4	1.06
Pm-M15	1331–1411	1372	15.4	1.12
Pm-M20	1297–1392	1314	26.1	1.99
Pm-M25	1259–1331	1299	14.1	1.09

**Table 4:** Results of bending strength testing of modified cement-bonded particleboards.

Mixture	Bending Strength [N/mm^2^]	Average Bending Strength [N/mm^2^]	Absolute Error [N/mm^2^]	Relative Error [%]
Ce-Ref	11.2–12.5	12.0	0.3	2.30
Ce-M10	11.1–12.2	11.7	0.2	1.92
Ce-M15	10.3–10.9	10.6	0.1	1.11
Ce-M20	8.9–10.4	9.8	0.3	3.12
Ce-M25	8.6–10.9	9.7	0.4	4.51
Du-Ref	11.2–12.2	11.7	0.2	1.73
Du-M10	9.8–13.1	11.4	0.6	5.63
Du-M15	10.5–11.5	10.9	0.2	1.83
Du-M20	9.3–11.4	10.4	0.4	3.95
Du-M25	8.9–11.6	10.3	0.5	5.10
Pm-Ref	9.9–12.4	11.6	0.5	4.33
Pm-M10	10.9–11.7	11.3	0.2	2.06
Pm-M15	10.6–12.2	11.4	0.3	2.51
Pm-M20	9.6–11.0	10.3	0.2	2.25
Pm-M25	8.9–12.1	10.5	0.6	5.91

**Table 5:** Results of modulus of elasticity in bending testing of modified cement-bonded particleboards.

Mixture	Modulus of Elasticity in Bending [N/mm^2^]	Average Modulus of Elasticity in Bending [N/mm^2^]	Absolute Error [N/mm^2^]	Relative Error [%]
Ce-Ref	7054–7881	7496	160.3	2.14
Ce-M10	5987–6774	6356	152.3	2.40
Ce-M15	6080–6382	6231	58.2	0.93
Ce-M20	5036–6174	5487	232.7	4.24
Ce-M25	3757–5847	4710	406.9	8.64
Du-Ref	7960–8250	8105	55.9	0.69
Du-M10	6119–7265	6692	220.5	3.30
Du-M15	5389–6874	6111	286.2	4.68
Du-M20	5032–7388	6374	466.3	7.32
Du-M25	3694–5653	4578	382.4	8.35
Pm-Ref	7730–8413	8160	139.9	1.71
Pm-M10	6870–7569	7220	134.5	1.86
Pm-M15	6715–7776	7246	204.2	2.82
Pm-M20	6045–6752	6399	136.0	2.13
Pm-M25	4021–5981	4965	377.9	7.61

**Table 6:** Results of transverse tensile strength perpendicular to the plane of the board testing of modified cement-bonded particleboards.

Mixture	Transverse Tensile Strength [N/mm^2^]	Average Transverse Tensile Strength [N/mm^2^]	Absolute Error [N/mm^2^]	Relative Error [%]
Ce-Ref	0.96–1.01	0.99	0.01	0.95
Ce-M10	0.67–0.79	0.75	0.03	3.39
Ce-M15	0.69–0.83	0.75	0.03	3.71
Ce-M20	0.62–0.78	0.72	0.03	4.66
Ce-M25	0.59–0.78	0.68	0.04	5.40
Du-Ref	0.86–0.89	0.88	0.01	0.73
Du-M10	0.76–0.92	0.83	0.03	3.80
Du-M15	0.61–0.81	0.71	0.04	5.42
Du-M20	0.44–0.64	0.54	0.04	7.13
Du-M25	0.35–0.57	0.49	0.05	9.56
Pm-Ref	0.84–0.97	0.91	0.02	2.63
Pm-M10	0.77–0.85	0.82	0.02	2.05
Pm-M15	0.65–0.81	0.74	0.03	4.26
Pm-M20	0.55–0.81	0.66	0.05	7.85
Pm-M25	0.38–0.71	0.57	0.07	11.52

## Data Availability

The datasets used and/or analysed during the current study are available from the corresponding author on reasonable request.
